# Fusion-independent virus-like particles enable rapid detection of antibody-mediated inhibition of ligand-receptor interactions

**DOI:** 10.3389/fimmu.2026.1943580

**Published:** 2026-10-28

**Authors:** Fangfeng Yuan, Jianzhu Chen

**Affiliations:** Koch Institute for Integrative Cancer Research and Department of Biology, Massachusetts Institute of Technology, Cambridge, MA, United States

**Keywords:** African swine fever virus, binding-inhibition, BSL-2, CD2v, ligand-receptor interactions, select agent diagnostics, virus-like particles

## Abstract

Studying antibody-mediated inhibition of ligand-receptor interactions is critical for understanding fundamental biological processes and developing therapeutic interventions. However, for high-consequence viruses with entry machinery involving multiple viral proteins, it is often difficult to develop pseudotyped viruses that can infect susceptible host cells. Here, we report a rapid platform for detecting antibody-mediated inhibition of ligand-receptor interactions by decoupling viral attachment from post-entry membrane fusion and gene expression. Our platform leverages HIV Gag-based self-assembling virus-like particles (VLPs) where Gag is fused with GFP and further pseudotyped with selected ligands for easy readout of VLP binding to susceptible cells. We first validated the platform’s stringent cell-type specificity using broadly tropic VSV-G and CD19-specific single chain fragment variable (scFv) pseudotypes. Next, we applied this technology to African swine fever virus (ASFV) - a high-consequence agricultural pathogen lacking surrogate neutralization assays due to its multi-protein entry machinery. By screening VLPs pseudotyped with different viral proteins against diverse mammalian cell lines, we found that VLP pseudotyped with ASFV CD2v binds specifically to Vero cells. Leveraging this interaction, we developed a rapid test for detecting antibodies that inhibit CD2v-pseudotyped VLP binding to Vero cells. Longitudinal analysis of sera from mRNA-LNP vaccinated pigs showed that the degrees of VLP-based binding-inhibition strongly correlated with the levels of anti-CD2v IgG, validating the assay’s utility. Overall, the pseudotyped VLP platform provides an enabling tool for ligand-receptor discovery, vaccine evaluation, and disease surveillance for select agents.

## Introduction

Ligand-receptor interaction studies are critical for understanding biological processes and pharmaceutical development. For viral infections, studying antibody-mediated inhibition of ligand-receptor interactions is fundamental to understanding viral infections and assessment of immune responses. Serological neutralization assays have been used to study such interactions. However, development of such assays for highly pathogenic viruses is constrained by two barriers: requirement for high biosafety containments and incomplete understanding of viral entry mechanisms. Live virus-based plaque reduction neutralization tests (PRNT) remain the gold standard but often require BSL-3/4 containment. Building on well-characterized viral entry mechanisms, lentivirus pseudotyped with viral attachment proteins or receptor binding proteins have been developed to deliver reporter genes into receptor-bearing cells to assay for neutralizing antibodies (nAbs) specific to the viral proteins (1). For example, the pseudovirus-based assay has been widely used for studying SARS-CoV-2 entry mechanism and vaccine evaluation (2). Similarly, ELISA-based receptor-binding inhibition assays can serve as a proxy for the measurement of antiviral nAbs (3). These tools have also been successfully used to evaluate nAbs against other viruses such as Influenza virus, Ebola virus (4–7).

In general, pseudoviruses require the displayed viral proteins to mediate both cellular attachment and membrane fusion. However, for high-consequence viruses with unknown entry mechanisms, such as African swine fever virus (ASFV), traditional pseudotyping approaches have not worked, creating a diagnostic gap that impedes vaccine development and outbreak response. ASFV is a large DNA virus (150–190 nm, ~170 kb genome) with complex virion architecture. Unlike most other enveloped viruses employing single or dual receptor-dependent entry pathways, ASFV utilizes multiple mechanisms including receptor-mediated endocytosis, clathrin-mediated endocytosis, macropinocytosis, and phagocytosis to infect host cells. Because multiple proteins are involved in viral fusion with host cells, traditional lentivirus pseudotyped with a single ASFV viral protein fail to achieve cellular entry and reporter gene expression. Consequently, no validated surrogate neutralization assay exists for ASFV, severely limiting vaccine development and outbreak response, particularly in endemic regions lacking high-containment facilities.

Based on HIV Gag-mediated self-assembly of virus-like particles (VLPs) (8) and the use of fluorescent VLP for studying HIV infection (9–11), we sought to overcome the limitations of pseudotyped lentiviruses by developing a receptor-agnostic, fusion-independent platform that decouples viral attachment from post-entry membrane fusion and reporter gene expression. Specifically, we fused the Gag protein with a GFP reporter and further pseudotyped VLP with different ASFV proteins for easy readout of VLP binding to susceptible cells. We validated this concept using the Vesicular Stomatitis Virus G protein (VSV-G), which has broad tissue tropism. By pseudotyping engineered VLPs with a membrane-bound single chain fragment variable (scFv) specific for CD19, we showed that scFv-pseudotyped VLPs bind efficiently and specifically to CD19-bearing B cells in a dose- and receptor density-dependent manner. Using ASFV as a model, we pseudotyped fluorescent VLPs with different viral membrane proteins, including CD2v, EP153R, P54, P22, E199L, E248R. We screened a series of cell lines to test the binding of pseudotyped VLPs and found that CD2v pseudotyped VLP bind specifically to Vero cells. Using this system, we evaluated the ability of sera from CD2v mRNA-lipid nanoparticle (LNP) immunized pigs to inhibit binding of CD2v pseudotyped VLP to Vero cells and found that the antibody-mediated inhibition correlated with the titer of anti-CD2v IgG. Overall, our study provides a novel strategy to assess antibody-mediated inhibition of ligand-receptor interactions for select agents in BSL-2 settings, facilitating vaccine evaluation and viral entry mechanism studies.

## Results

### Engineered fluorescent VLPs enable rapid, receptor-specific binding detection independent of viral transduction

Traditional pseudovirus assays require the viral envelope protein to mediate both cellular attachment and membrane fusion to deliver a reporter gene into the host cell. To bypass the requirement for membrane fusion, which is often intractable for select agents with complex or unknown entry mechanisms, we engineered a non-infectious, fluorescent Virus-Like Particle (VLP) platform. By fusing enhanced green fluorescent protein (eGFP) directly to the HIV-1 Gag protein, we generated self-assembling VLPs that are intrinsically fluorescent, decoupling the measurement of receptor binding from post-attachment viral entry events (**Figure 1A**).

**Figure 1:**
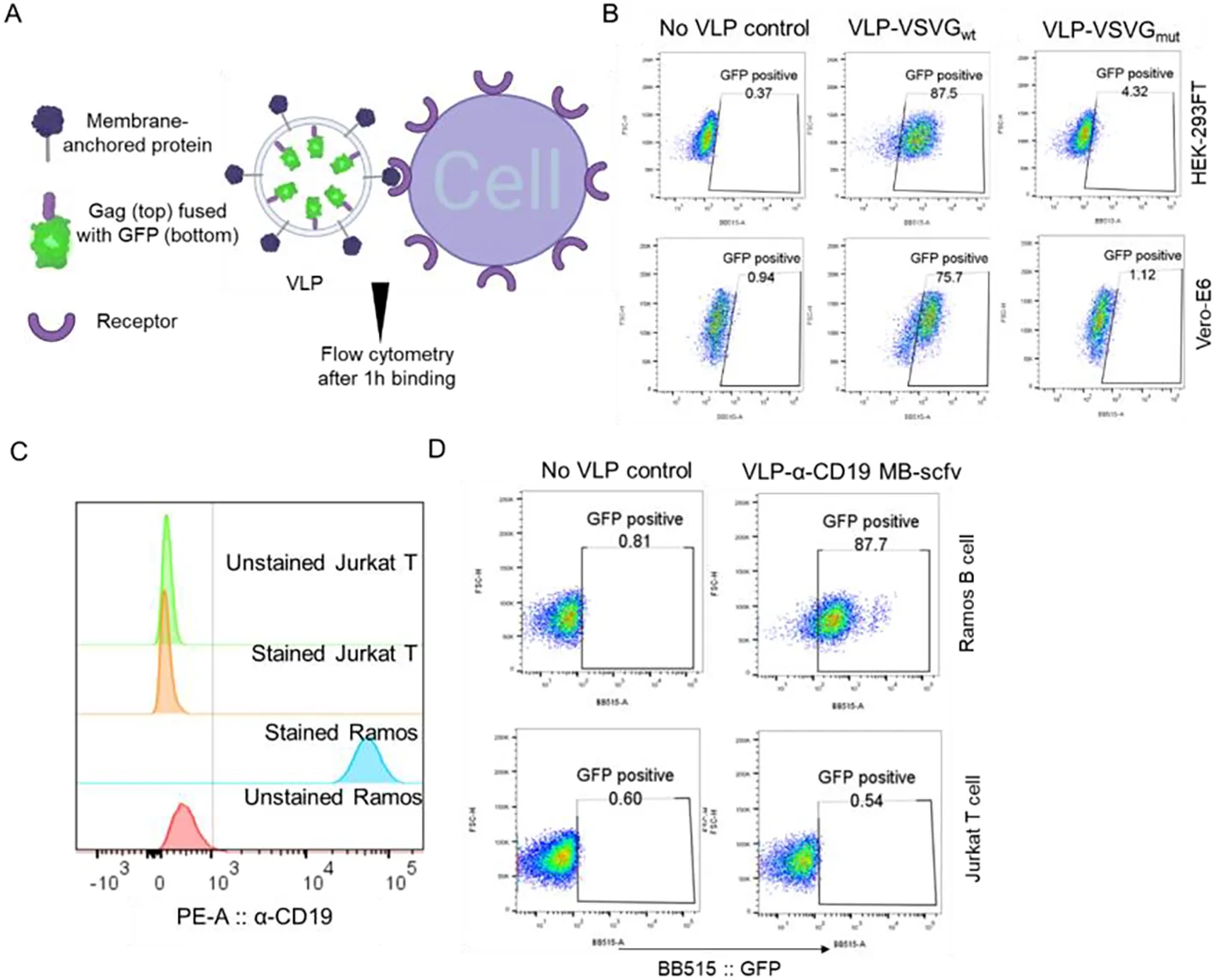
Engineered Gag-eGFP VLPs enable receptor-specific binding independent of entry and reporter gene expression. **(A)** Schematic diagram illustrating the interaction between a surface ligand pseudotyped fluorescent VLP and a receptor-bearing host cell. Binding is quantified at 1 hour post-incubation via flow cytometry for eGFP-positive cells. **(B)** Representative flow cytometry plots showing the binding of wild-type VSV-G pseudotyped VLPs (VLP-VSVG_wt_) to HEK-293FT (Top) and Vero-E6 (Bottom) cells. VLPs pseudotyped with a receptor binding-deficient mutant VSV-G (VLP-VSVG_mut_) showed abolished most binding activity. **(C)** Flow cytometry plots showing CD19 surface expression on human Ramos B cells but not on Jurkat T cells following anti-CD19 antibody staining. **(D)** Specific binding of scFv-pseudotyped VLPs to CD19-positive Ramos B cells (Top) but not Jurkat T cells (Bottom).

To validate the specificity of this platform, we first pseudotyped the Gag-eGFP VLPs with the Vesicular Stomatitis Virus G protein (VSV-G), known for its ubiquitous cell tropism. As expected, VLPs displaying wild-type VSV-G (VLP-VSVG_wt_) bound efficiently to both human HEK-293FT and monkey Vero-E6 cells. In contrast, VLPs displaying a mutant VSV-G (VLP-VSVG_mut_), where K37Q/R354Q mutations disrupt binding to low-density lipoprotein receptor (LDLR), did not exhibit any detectable binding (**Figure 1B**). To further demonstrate that this platform can measure highly specific, cell-type dependent interactions, we pseudotyped the VLPs with a membrane-bound scFv specific for human CD19 (VLP-α-CD19 MB-scFv). Flow cytometry analysis revealed that VLP-α-CD19 MB-scFv bound specifically and robustly to human B cell line Ramos but not human T cell line Jurkat (**Figures 1C, D**). These data establish that Gag-eGFP VLPs pseudotyped with surface ligands can report specific interactions between the ligands and cellular receptors.

### Optimization of VLP binding conditions improve ligand-receptor specificity

To maximize the signal-to-noise ratio of the VLP binding assay, we evaluated several variables known to influence viral attachment, including the use of electrostatic-reducing agents, VLP dosage, and cell density. Polybrene is widely used in traditional lentiviral transductions to neutralize charge repulsions between virions and the cell surface. However, the addition of 8 µg/mL polybrene dramatically increased cell-associated fluorescence, including signal detected with VLP-VSVG_mut_ (**Figure 2A**). Because polybrene reduced the ability of the assay to distinguish receptor-dependent binding from background particle association, it was excluded from all subsequent experiments to maximize assay specificity.

**Figure 2:**
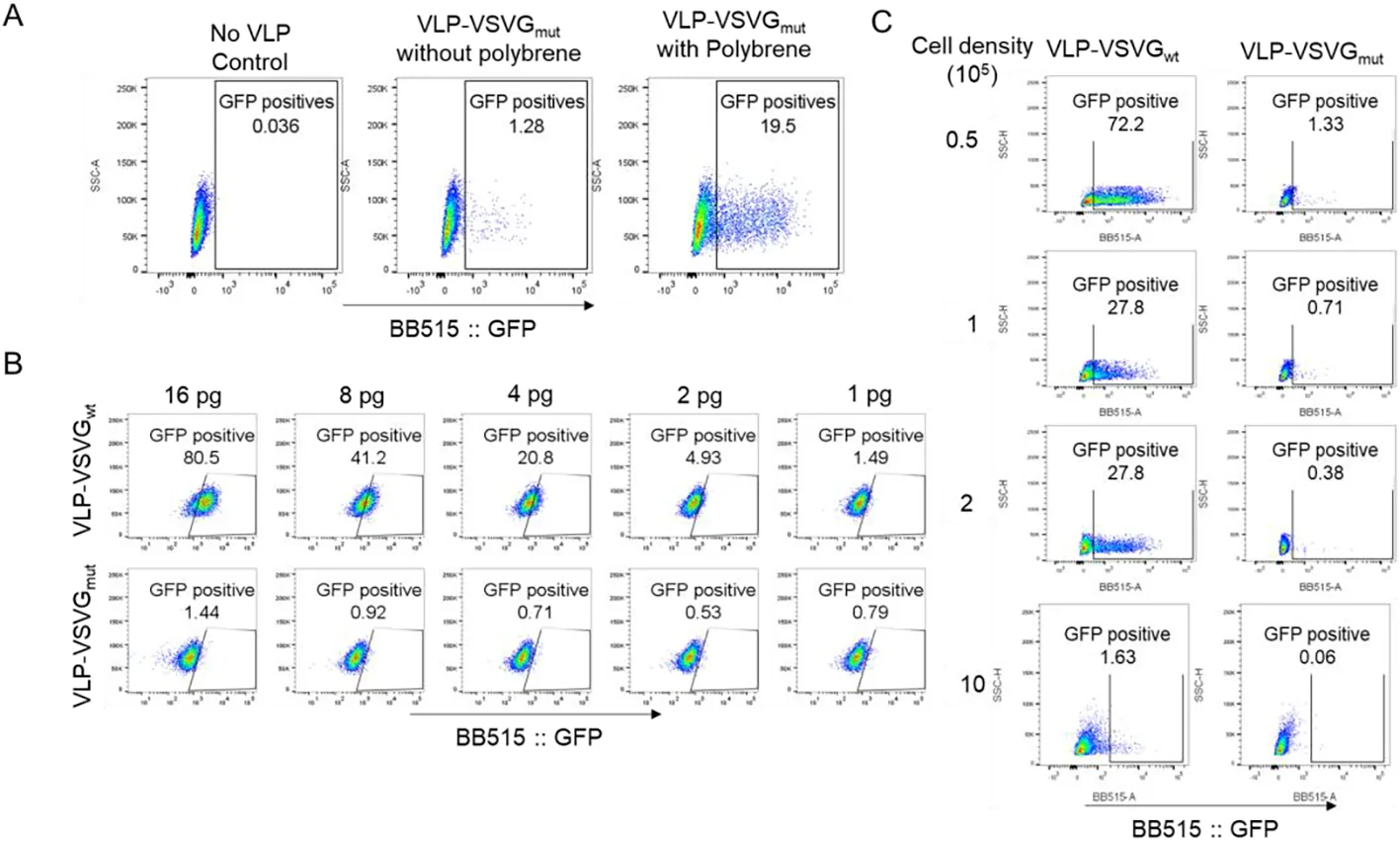
Optimization of VLP assay conditions reveals critical parameters for binding specificity. **(A)** Impact of electrostatic-reducing agents on assay specificity. 10 pg of VLP-VSVG_wt_ or VLP-VSVG_mut_ were incubated with HEK-293FT cells in one well of 96-well plate for 1 hour in the presence or absence of 8 µg/mL polybrene, followed by washing and flow cytometric analysis. **(B)** The effect of VLP dose on target cell binding. VLP-VSVG_wt_ and VLP-VSVG_mut_ (1 pg to 16 pg) were incubated with 0.5 ×10^5^ cells in a 96-well plate for one hour followed by flow cytometry analysis. **(C)** The effect of host cell seeding density on VLP binding efficiency. HEK-293FT cells (from 10^6^ to 0.5 ×10^5^ cells/well) were incubated with equal amounts (16 pg) of VLP-VSVG_wt_ or VLP-VSVG_mut_ for 1 hour. For all panels, the GFP-positive cell populations were gated and quantified.

We next assessed VLP dosing and host cell density. Binding of VLP-VSVG_wt_ to HEK-293FT cells occurred in a strict dose-dependent manner, whereas VLP-VSVG_mut_ remained at baseline across all tested doses (**Figure 2B**). Furthermore, optimizing the cell seeding density was critical; binding efficiency peaked at lower cell densities and inversely correlated with over-confluent cell populations (**Figure 2C**), likely due to reduced receptor availability or steric hindrance in densely packed cell cultures. These optimized conditions (no polybrene, titrated VLP dose, and sub-confluent cell densities) are used for all downstream assay development.

### A VLP-based screen identifies specific binding of ASFV CD2v to Vero cells

ASFV entry and membrane fusion is a multi-protein coordinated process that is not well-understood. The virion possesses multiple membrane proteins (e.g., CD2v, EP153R, P22, E199L, E248R, P54) involved in attachment, macropinocytosis, and endosomal fusion (**Supplementary Figure 1A**). We first attempted to develop a traditional lentivirus-based neutralization assay by pseudotyping lentivirus with individual ASFV membrane proteins. The pseudotyped lentiviruses were incubated with a panel of ASFV-susceptible cell lines (Vero-E6 from the kidney of an African green monkey, 3D4/31 from porcine alveolar macrophages, PAOEC from porcine aortas, PT-K75 from porcine nasal turbinate mucosa) for 48 hours (**Supplementary Figure 1B**). While the positive control (VSVG_wt_-lentivirus) successfully transduced all tested cell lines, yielding robust ZsGreen expression, none of the lentiviruses pseudotyped with ASFV proteins (CD2v, EP153R, P22, P54, E248R, or E199L) resulted in any significant ZsGreen expression (**Supplementary Figure 1C**).

We next determined whether the fluorescent VLP can be pseudotyped with ASFV proteins and used for assaying VLP binding to susceptible cells (**Figure 3A**). We pseudotyped Gag-eGFP VLPs with individual ASFV membrane proteins CD2v, EP153R, P22, P54, E248R, and E199L with a HA tag at the C-terminal. Incorporation of ASFV protein into VLP was verified by anti-HA Western blotting (WB) of purified VLPs (**Supplementary Figure 2**).

**Figure 3:**
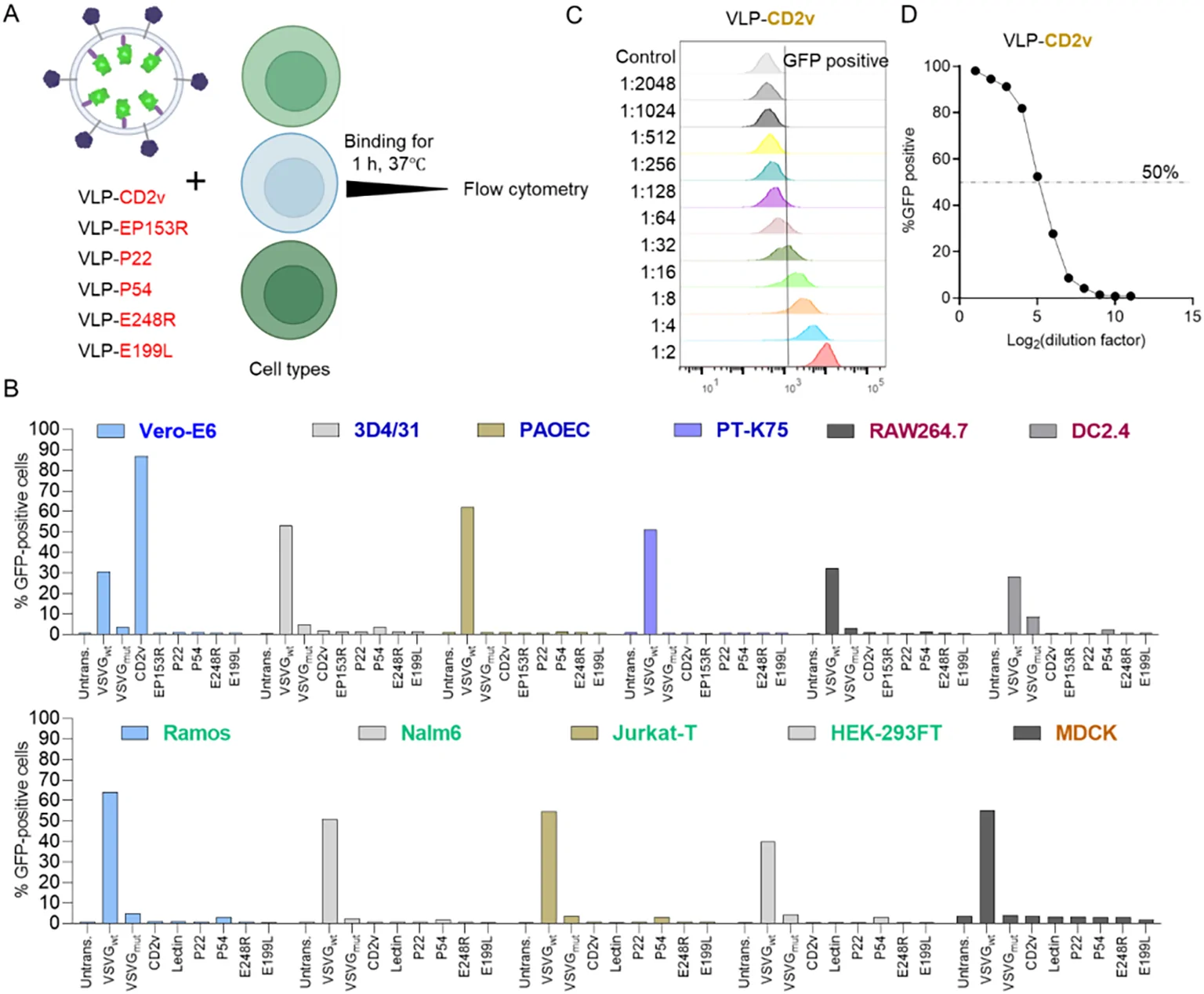
ASFV CD2v pseudotyped VLP binds specifically and robustly to Vero-E6 cells. **(A)** Experimental workflow for the VLP binding assay. Fluorescent Gag-eGFP VLPs pseudotyped with individual ASFV proteins were incubated with a panel of mammalian cell lines for 1 hour prior to flow cytometric analysis of GFP-positive cells. **(B)** Bar graphs showing the percentage of cells positive for VLP binding across the indicated cell lines. **(C)** Dose-dependent binding of VLP-CD2v to Vero-E6 cells. The GFP-positive population was gated relative to no VLP control cells. **(D)** Binding curve of VLP-CD2v to Vero-E6 cells. The dashed line indicates the 50% maximal GFP-positive binding threshold within the exponential phase of the curve.

We tested binding of the ASFV protein-pseudotyped VLPs to 11 mammalian cell lines derived from diverse cell lineages, including macrophages, endothelial cells, and epithelial lineages, as well as cell types known to be susceptible to ASFV infection, including Vero-E6, 3D4/31, PAOEC and PT-K75. While most VLPs showed no significant binding to the panel of cell lines, VLPs pseudotyped with the CD2v (EP402R) exhibited robust binding to the Vero-E6 cells (**Figure 3B**). In contrast, much higher doses of VLP-P54 (10–160 pg) did not show any significant binding to Vero-E6 cells (**Supplementary Figure 3**). Furthermore, VLP-CD2v binding to Vero-E6 exhibited a clear dose-dependence with a rapid binding kinetics (**Figures 3C, D**) The dose rendering 50% binding (exponential phase of the curve) was selected for downstream assay development as it maximizes the assay sensitivity.

### The VLP-based assay can be used to quantify anti-CD2v antibody-mediated inhibition of VLP-CD2v binding to Vero-E6 cells

To demonstrate the utility of the VLP-based binding assay as a serological diagnostic tool, we developed a VLP-CD2v based binding-inhibition test (**Figure 4A**). We utilized longitudinal serum samples from pigs immunized with CD2v mRNA-LNP vaccine (12). Immune sera were incubated with VLP-CD2v prior to incubation with Vero-E6 cells, and binding inhibition was quantified via flow cytometry (**Figure 4B**).

**Figure 4:**
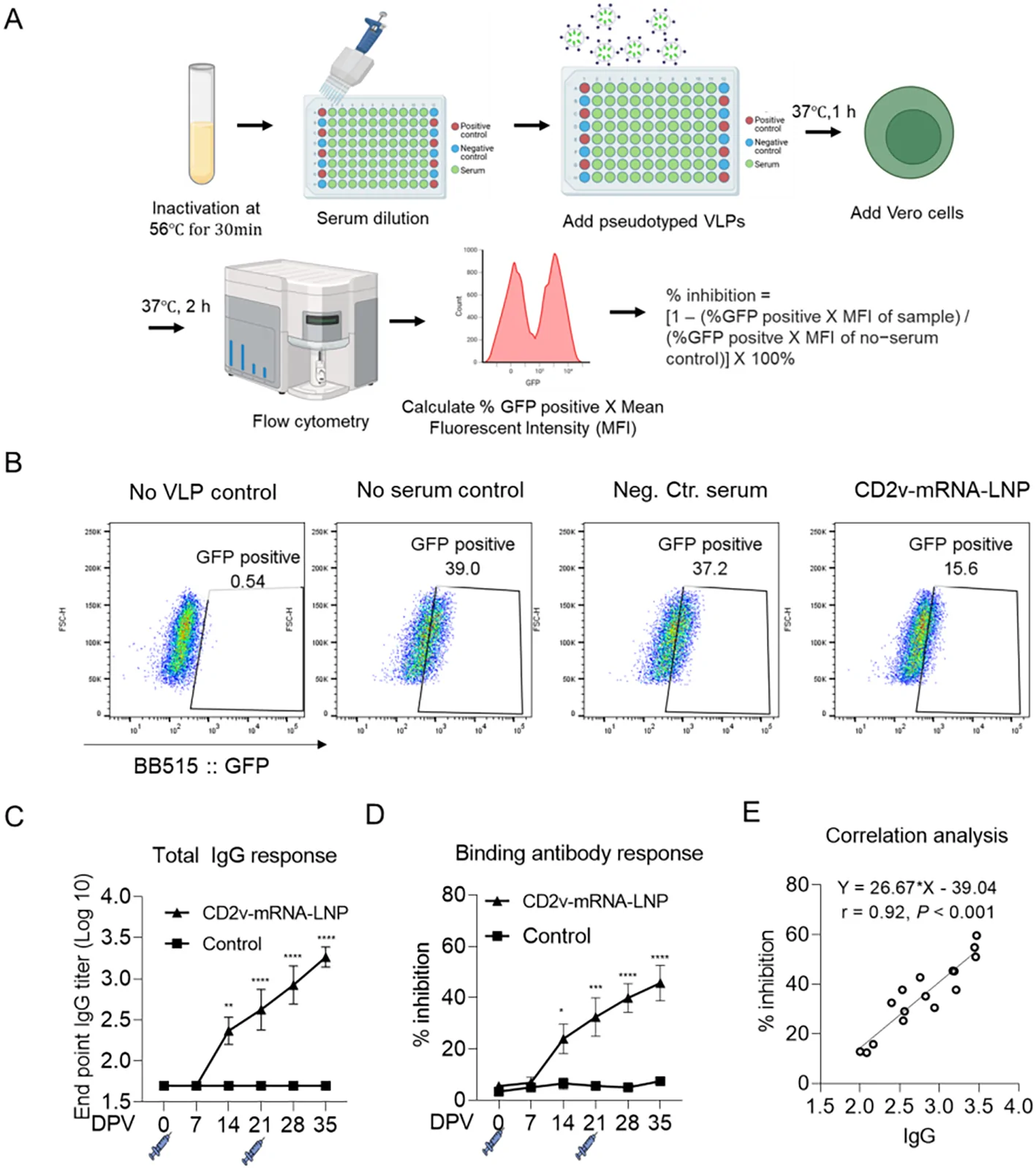
The VLP-CD2v assay accurately quantifies vaccine-induced antibody responses. **(A)** Schematic workflow of the VLP-CD2v based binding inhibition assay. **(B)** Representative flow cytometry plots depicting VLP-CD2v binding inhibition in the presence of serum collected from CD2v-mRNA-LNP immunized pigs. Controls include no-VLP control, a no-serum control, and a pre-immune negative serum control. **(C)** Longitudinal total anti-CD2v IgG responses measured by indirect ELISA in pigs immunized with CD2v-mRNA-LNP or a PBS control. **(D)** Longitudinal CD2v-specific antibody response quantified via the VLP-CD2v binding inhibition assay. **(E)** Pearson correlation analysis showing a strong positive relationship between the percentage of VLP-CD2v binding inhibition and total anti-CD2v IgG endpoint titers. **(C–E)** Four pigs of each group were immunized on day 0, boosted on day 21. Data shown here **(C, D)** are from all the animals within each group and represented as mean ± SEM. Statistical significance was determined using one-way ANOVA with appropriate multiple-comparison tests. Groups showing statistical difference are denoted with asterisks ^*^P < 0.05, ^**^P < 0.01, ^***^P < 0.001, and ^****^P < 0.0001.

We directly compared total anti-CD2v IgG titers as measured by traditional ELISA to antibody-mediated inhibition as measured by VLP-CD2v assay. Total CD2v-specific IgG became detectable 14 days following primary immunization and increased steadily following boost at day 21 (**Figure 4C**). Similarly, anti-CD2v antibody-mediated inhibition became evident at day 14 and increased steadily following boost (**Figure 4D**). Pearson correlation analysis revealed a strong, positive correlation (r=0.92) between the percentage of VLP-CD2v binding inhibition and total anti-CD2v IgG titers (**Figure 4E**). These results show that the VLP-based assay can quantify antibody responses to specific antigen and therefore can serve as a functional surrogate for monitoring vaccine-induced attachment-blocking antibodies.

## Discussion

In this study, we established a modular platform for the rapid assessment of antibody-mediated inhibition of ligand-receptor interactions for select agents. By exploiting the inherent ability of HIV Gag to self-assemble into virus-like particles and fusing a reporter directly to Gag, we bypassed the hurdle in traditional pseudovirus technology: the requirement for membrane fusion and subsequent reporter gene expression. This platform exhibited three major advantages over traditional pseudovirus-based test.

First, the VLP-based assay has a faster turnaround time. Standard lentiviral pseudovirus neutralization tests - such as those widely used for SARS-CoV-2 or Influenza - require 48 to 72 hours of incubation for the expression of reporter gene (e.g., luciferase or GFP). In contrast, because our engineered VLPs are intrinsically fluorescent, the binding-inhibition assay directly measures physical virion attachment. This reduces the entire experimental turnaround time from days to just a few hours.

Second, this platform is uniquely suited for viruses with complex entry mechanisms. The failure of lentiviruses pseudotyped with individual ASFV antigens to transduce susceptible cells highlights the limitations of applying pseudoviruses to complex viruses. ASFV utilizes a highly complex, multi-step entry mechanism involving macropinocytosis and endocytosis, followed by outer envelope disruption and inner envelope fusion driven by a multi-protein mediated mechanism (e.g., E199L, E248R). Pseudovirus displaying a single ASFV protein have not found to orchestrate this endosomal escape of reporter gene after viral entry. By reducing the entry equation strictly to the initial attachment event, our VLP platform quantifies attachment-blocking antibodies rather than reporter gene expression after membrane fusion.

Third, and perhaps most importantly, the VLP platform is fundamentally receptor-agnostic. For many select agents and newly emerging viruses, the specific cellular receptors are unknown or highly redundant. Traditional pseudovirus systems often rely on overexpression of an identified receptor on a cell line to render infection which includes receptor engagement, endocytic trafficking, membrane fusion, and cytoplasmic delivery of the reporter gene. With the VLP platform, researchers do not need to know the identity of the host receptor of select agents; they only need an empirically susceptible cell line. The observation that CD2v-pseudotyped VLPs bound efficiently to Vero-E6 cells while CD2v-pseudotyped lentiviruses failed to transduce these cells suggests that CD2v primarily contributes to viral attachment rather than mediating the complete entry process. This observation strongly suggests the presence of a specific, yet-to-be-identified receptor enriched on Vero cells (potentially shared with erythrocytes given CD2v’s role in hemadsorption). However, Vero cells are not natural ASFV target cells, and the cellular factor responsible for this interaction may not represent a physiologically relevant ASFV receptor. Future studies will be required to determine whether the same cellular factor is present in porcine target cells and whether it contributes to ASFV attachment, tropism, or pathogenesis. Overall, our platform serves as an immediate diagnostic workaround, while also acting as a tool to facilitate the receptor discovery.

The potential application of this platform was validated using serum from pigs immunized with a CD2v mRNA-LNP vaccine, demonstrating a robust correlation between total IgG and binding-inhibitory antibody titers. It should be noted that the current study utilized vaccine-induced sera; future studies must validate this platform using convalescent serum from ASFV-infected swine to evaluate how the natural poly-antigen immune response inhibit binding. Because this platform exclusively measures attachment rather than full viral entry, future studies utilizing homologous live ASFV strains in BSL-3 containment are required to definitively correlate these VLP binding-inhibition titers with true live-virus neutralization. Because ASFV protection is likely polygenic, evaluating antibodies against a single protein may not fully predict *in vivo* protection.

Compared to recombinant protein ELISAs which rely on recombinant proteins that often lack transmembrane domains, the VLP assay displays viral proteins in their native lipid-bilayer conformation and provides a functional readout rather than merely detecting protein-binding. However, the VLP assay relies on cell cultures and requires flow cytometry infrastructure. Adaptation to ultra-high-throughput automation is needed in the future.

In conclusion, our Gag-eGFP VLP platform circumvents the strict biosafety requirements and biological barriers that have historically impeded select agent research. By providing a safe, rapid method for binding-inhibition detection in BSL-2 settings, this platform may facilitate disease surveillance, vaccine evaluation, receptor discovery, and studies of viral tropism.

## Materials and methods

### Cell lines and culture conditions

The human embryonic kidney cell line HEK-293FT, African green monkey kidney cell line Vero-E6, Madin-Darby canine kidney cell line MDCK, murine macrophage cell line RAW264.7, and porcine turbinate cell line PT-K75 were maintained in Minimum Essential Medium (MEM). Media were supplemented with 10% fetal bovine serum (FBS), penicillin (100 U/mL), streptomycin (100 µg/mL), and amphotericin B (fungizone, 0.25 µg/mL). Cells were cultured at 37 °C in a humidified incubator with 5% CO_2_. The porcine alveolar macrophage cell line 3D4/31, porcine aortic endothelial cell line PAOEC, mouse dendritic cell line DC2.4, human B lymphocyte cell line Ramos, human B cell precursor leukemia cell line Nalm6, and human T lymphocyte cell line Jurkat were maintained in RPMI-1640 medium supplemented with 10% FBS, antibiotics, and fungizone as described above.

### WB and VLP purification

All primers were synthesized by IDT DNA Technologies, and plasmid constructs were generated using Gibson Assembly (New England Biolabs). The pMD2.G vector was used to express wild-type VSV-G (13). A pMD2.G vector expressing a double-mutant VSV-G (K37Q, R354Q) lacking receptor-binding activity, alongside a pMD2.G-CD19scFv construct, were graciously provided by Dr. Michael Birnbaum’s laboratory at MIT. To generate intrinsically fluorescent VLPs, the eGFP gene was fused in-frame to the HIV-1 gag gene using standard cloning procedures. Sequences encoding ASFV membrane proteins—CD2v (EP402R), EP153R (C-type lectin), P22 (KP177R), P54 (E183L), E248R, and E199L—derived from the Georgia 2007/1 strain (NCBI Reference Sequence: NC_044959.2) were synthesized by GenScript and subcloned into the phCMV expression vector.

To produce VLPs, HEK-293FT cells were seeded in 150-mm dishes 24 hours prior to transfection. Cells were co-transfected with 20 µg of the target membrane protein expression plasmid and 30 µg of the Gag-eGFP plasmid using linear polyethylenimine (PEI, MW 40,000; Polysciences). Culture media was refreshed 8 hours post-transfection. Supernatants containing VLPs were harvested at 24 and 72 hours post-transfection, pooled, and centrifuged at 800 × g for 10 minutes to remove cell debris. Cleared supernatants were passed through a 0.45-µm filter (Millipore) and concentrated using PEG-it Virus Precipitation Solution (System Biosciences) according to the manufacturer’s instructions. VLP pellets were resuspended in 1 mL of sterile PBS, aliquoted, and stored at -80 °C until use. To quantify the VLPs, we used a commercial HIV-1 p24 antigen ELISA kit (XpressBio) according to the manual. The concentration (pg/mL) of VLPs was determined through a standard curve using serial dilutions of standards included in the kit.

For WB analysis of purified VLPs, 20 μL per sample were boiled in loading buffer, resolved by SDS-PAGE, and transferred onto a nitrocellulose membrane. Membranes were incubated with a rabbit polyclonal antibody specific for the hemagglutinin (HA) epitope tag, followed by detection using StarBright™ Blue 700-conjugated goat anti-rabbit IgG (Bio-Rad). Fluorescent signals were visualized and recorded using a ChemiDoc™ MP Imaging System (Bio-Rad Laboratories). The calculated molecular weight is 23.4 kDa for E199L, 21.4 kDa for P22, 28.8 kDa for E248R, 19.8 kDa for EP153R, 21.4 kDa for P54, 42.2 kDa for CD2v.

### Optimization of VLP-cell binding conditions

For VLP binding assay, purified VLP was incubated with 50,000 cells in 100 µL in one well of a 96-well plate at 37 °C for 1 h. Cells were centrifuged at 800 g for 10min and washed for three times using DAPI-containing PBS for flow cytometry to quantify GFP positive cells. For testing the effect of polybrene on binding activity, purified VLP-VSVG_mut_ was mixed with or without 8 µg/ml polybrene (Millipore) before incubation with HEK-293FT cells. For testing the dose-dependent effect, a two-fold serial amount (1pg to 16pg) of VLP-VSVG_wt_ and VLP-VSVG_mut_ was used to incubate with HEK-293FT cells in a 96-well tissue culture plate. For testing the effect of different cell densities on binding activity, a 2-fold serial of HEK-293FT cell densities from 0.5×10^5^ to 10×10^5^ were incubated with VLP-VSVG_wt_ and VLP-VSVG_mut_ in a 96-well tissue culture plate at 37 °C for 1 h before flow cytometry analysis.

### ASFV pseudotyped lentivirus production and infection

To generate conventional ASFV pseudotyped lentivirus, HEK-293FT cells were seeded into 150 mm dishes and transfected with the pCMV antigen expression vector (10 µg), packaging vector psPAX2 (20 µg), and transfer vector pLVX-ZsGreen (20 µg) using Linear Polyethylenimine (PEI, MW 40,000) (Polysciences) as described previously (14). The media was changed in 8 hours post-transfection, and viral supernatants were collected at 48 and 72 hours. Collected supernatants were spun down at 4,300 rpm for 10 minutes and filtered through a 0.45-µm SFCA filter (Thermo Scientific), followed by 60-fold concentration using PEG-it Virus Precipitation Solution (System Biosciences) according to the manufacturer’s protocols. Lentiviruses were resuspended in 1 mL sterile PBS, aliquoted, and stored at -80 °C until use.

For lentiviral infection, target cells were seeded in 96-well plates at a density of 20,000 cells in 100 µL per well and transduced with purified pseudotyped lentivirus. Cells were trypsinized in 48 hours post-transduction and washed for three times. GFP signal was measured by flow cytometry.

### Serum samples and assay evaluation

Pig serum samples were obtained in a previous study (12). Briefly, four-week-old piglets were immunized intramuscularly with 30 µg of LNP-mRNA expressing CD2v or PBS control, and boosted after three weeks. Serum samples were collected on days 0, 7, 14, 21, 28, and 35 post immunization.

Indirect ELISA. For detection of CD2v antibody response in serum from immunized animals, 300 ng purified proteins were coated onto 2HB ELISA plate (Thermo Fisher) in antigen coating buffer (35 mM sodium bicarbonate and 15 mM sodium carbonate, pH 8.8) and incubated at 37 °C overnight (15). Cutoff values for each protein were determined by OD value of control group animal samples plus three standard deviations. The endpoint titer for each animal that has the highest dilution giving a reading above cutoff was calculated by interpolating from a sigmoidal standard curve using GraphPad Prism.

Pseudotyped VLP-based binding-inhibition test. To determine the attachment-blocking antibodies against ASFV CD2v, serum samples from LNP-mRNA immunized pigs were cleared by centrifuging at 10,000 rpm for 10 min in 4 °C and heat-inactivated at 56 °C for 30 min. Heat-inactivated serum samples were incubated with 10 pg (1:32 dilution of stock) VLP-CD2v at 37 °C for 1 hour. 200,000 corresponding cells were added to the wells and incubated for another hour at 37 °C. Cells were spun down and subjected to flow cytometry. The percentage of binding-inhibition was calculated using the formula: [1 – (%GFP positive × MFI of sample)/(%GFP positive × MFI of no−serum control)] × 100%.

### Statistical analysis

All the histogram charts were expressed as means +/- standard error of mean (SEM). Unless otherwise indicated, each *in vitro* experiment was performed independently at least three times with similar results, and representative data are shown. The number of biological replicates (n) is indicated in the figure legends where applicable. Differences between groups were evaluated by one-way analysis of variance (ANOVA) using Prism software version 6 (GraphPad Software). A *P* value of <0.05 was regarded as statistically significant difference.

## Data Availability

The original contributions presented in the study are included in the article/**Supplementary Material**. Further inquiries can be directed to the corresponding authors.
