# Supplementary material for: Fusion-independent virus-like particles enable rapid detection of antibody-mediated inhibition of ligand-receptor interactions
Source: Front Immunol. 2026 Oct 28;17:1943580. doi: 10.3389/fimmu.2026.1943580 (PMC13752075; doi:10.3389/fimmu.2026.1943580)
Supplement: Supplementary file 1 [file DataSheet1.docx]

 **Supplemental figure S1. Traditional ASFV-pseudotyped lentiviruses fail to transduce susceptible host cells.** (A) Schematic representation of the ASFV virion architecture. Key membrane and structural proteins (CD2v, EP153R, P22, E199L, E248R, P54) selected for pseudotyping are highlighted. (B) Experimental workflow for the traditional lentiviral transduction assay. Lentiviruses pseudotyped with individual ASFV membrane proteins were incubated with diverse ASFV-susceptible cell lines for 48 hours to allow for potential entry and reporter gene (ZsGreen) expression. (C) Bar graph depicting the percentage of ZsGreen-positive (transduced) cells. The X-axis represents lentiviruses pseudotyped with VSVGwt (positive control), ASFV CD2v, EP153R, P22, P54, E248R, or E199L. Panels represent data from Vero-E6, 3D4/31, PAOEC, and PT-K75 cell lines.

**Supplemental figure S2. Western blot analysis of ASFV proteins in purified VLPs.** Purified VLP preparations were subjected to SDS-PAGE followed by Western blot analysis using an anti-HA monoclonal antibody (mAb). The protein ladder is indicated on the left, and the protein bands shown include EP153R (19.8 kDa), CD2v (42.2 kDa), P54 (21.4 kDa), P22 (21.4 kDa), E199L (23.4 kDa), and E248R (28.8 kDa). The EP153R, CD2v are considered as glycosylated proteins.

**Supplemental figure S3. Dose-dependent testing of VLP-P54 binding with Vero-E6 cells.** VLP-P54 particles ranging from 10 to 160 pg (p24 equivalent) were incubated with Vero-E6 cells. After three washes to remove unbound particles, cell-associated fluorescence was quantified by flow cytometry to assess VLP-P54 binding. Corresponding percentage of positive cells are shown.
